# Palmatine Protects PC12 Cells and Mice from Aβ25-35-Induced Oxidative Stress and Neuroinflammation via the Nrf2/HO-1 Pathway

**DOI:** 10.3390/molecules28247955

**Published:** 2023-12-05

**Authors:** Yu Wang, Hongyan Pei, Weijia Chen, Rui Du, Jianming Li, Zhongmei He

**Affiliations:** 1 College of Chinese Medicinal Materials, Jilin Agricultural University, Changchun 130118, China; wangyu102629@163.com (Y.W.); phy19990505@163.com (H.P.); chenweijia_jlau@163.com (W.C.); durui@jlau.edu.cn (R.D.); 2 Key Laboratory of Animal Production, Product Quality and Security, Ministry of Education, Jilin Agricultural University, Changchun 130118, China

**Keywords:** Aβ25-35-induced Alzheimer’s disease, palmatine, Nrf2/HO-1 pathway, oxidative stress, neuroinflammation

## Abstract

Alzheimer’s disease is a common degenerative disease which has a great impact on people’s daily lives, but there is still a certain market gap in the drug research about it. Palmatine, one of the main components of Huangteng, the rattan stem of *Fibraurea recisa* Pierre (Menispermaceae), has potential in the treatment of Alzheimer’s disease. The aim of this study was to evaluate the neuroprotective effect of palmatine on amyloid beta protein 25–35-induced rat pheochromocytoma cells and AD mice and to investigate its mechanism of action. CCK8 assays, ELISA, the Morris water maze assay, fluorescent probes, calcein/PI staining, immunofluorescent staining and Western blot analysis were used. The experimental results show that palmatine can increase the survival rate of Aβ25-35-induced PC12 cells and mouse hippocampal neurons, reduce apoptosis, reduce the content of TNF-α, IL-1β, IL-6, GSH, SOD, MDA and ROS, improve the learning and memory ability of AD mice, inhibit the expression of Keap-1 and Bax, and promote the expression of Nrf2, HO-1 and Bcl-2. We conclude that palmatine can ameliorate oxidative stress and neuroinflammation produced by Aβ25-35-induced PC12 cells and mice by modulating the Nrf2/HO-1 pathway. In conclusion, our results suggest that palmatine may have a potential therapeutic effect on AD and could be further investigated as a promising therapeutic agent for AD. It provides a theoretical basis for the development of related drugs.

## 1. Introduction

Alzheimer’s disease (AD), a neurological disorder that occurs in old age and early old age, is highly prevalent and can be irreversible. Its common features include memory impairment, decreased thinking ability, impaired language function and behavioral disorders [1]. In recent years, the prevalence of AD has been on the rise, and could reach 13.8 million by 2050 [2]. The pathogenesis of AD is still not completely understood, and its incidence may be related to heredity, environment, or lifestyle. Studies have shown that the main pathological features of AD are extracellular amyloid β (Aβ) peptide plaques as well as intra-neural tau lesions [3]. Currently, the U.S. Food and Drug Administration (FDA)-approved drugs for the treatment of AD are cholinesterase inhibitors (ChEIs) and N-methyl-D-aspartate (NMDA) antagonists [4]. However, these are single-agent drugs that do not prevent or reverse the disease, and instead only partially alleviate the symptoms, and there is a risk of side effects. Therefore, there is an urgent need to find more effective drugs for AD [5,6].

Chinese medicine is multi-component, multi-targeted and modulates multiple signal pathways [7]. Studies have shown that traditional Chinese medicine may slow cognitive decline in AD patients by modulating Aβ production and aggregation, tau phosphorylation, and the gut microbiota–brain axis [8]. Up to now, there have been a lot of reports about the superiority of herbal drugs for treating AD as compared with single drugs. Huangteng is the rattan stem of *Fibraurea recisa* Pierre (Menispermaceae). Huangteng has been used medicinally in China for thousands of years and was first recorded in the Compendium of Materia Medica and also in the National Pharmacopoeia of the People’s Republic of China [9,10]. It can be used to treat diseases such as dysentery, hypertension, and inflammation, and is also indicated for the treatment of skin diseases [11,12]. Palmatine, a member of the protoberberine class of isoquinoline alkaloids, is a structural analog of berberine [13]. Palmatine is an alkaloidal component of huangteng, and has a variety of pharmacological effects [9,14,15]. For example, it attenuates doxorubicin-induced inflammatory response, oxidative damage, and cardiomyocyte apoptosis [16]; attenuates D-galactosamine-induced (D-gal) fulminant hepatic failure in mice [17]; and attenuates lipopolysaccharide-induced depressive-like behavior [18]. Palmatine crosses the blood–brain barrier and plays a role in the treatment of brain-related diseases [19,20]. In addition, palmatine has potent effects in reducing neuroinflammation and inhibiting acetylcholinesterase activity [19,21], which coincides with the pathogenesis of AD, and therefore palmatine may be a potential class of anti-AD drug candidates. We anticipate that palmatine may play a role in anti-inflammatory, antioxidant, and antiapoptotic aspects, thus slowing down the progression of AD development, and we hope that it will provide a new option for patients.

Rat pheochromocytoma cells (PC12) have neuronal characteristics and are similar to neurons in their cell morphology, structure, and function, and can easily be used in cultures due to their stable growth conditions [22]. Currently, they are commonly used as cell culture models for the protection against and repair of neurodegenerative diseases [23,24].

PC12 rat adrenal pheochromocytoma cells have been used as a cell model, and amyloid beta protein 25-35 (Aβ25-35) has been frequently used as a damage agent in AD research [25,26,27]. The deposition of Aβ has been mentioned as one of the etiological factors of AD [22,28]. Aβ25-35 is characteristic of elderly people and it is one of the most neurotoxic forms of Abeta. It is the shortest fragment capable of forming large β-sheet fibrils and retaining the toxicity of the full-length Aβ (1-40/42) peptides [29]. Aβ25-35 induces oxidative damage in PC12 cells, increasing intracellular ROS production and decreasing mitochondrial membrane potential, as well as decreasing cell viability, leading to apoptosis [30,31]. Notably, the injection of the Aβ25-35 fraction caused an increased NO level in the temporal cortex of rats, which likewise promotes the development of AD [32]. Experiments were conducted to investigate whether palmatine is protective against Aβ25-35-induced cell damage in PC12.

Oxidative stress injury plays an important role in the pathogenesis of AD and is associated with Aβ neurotoxicity [33]. Transcription factor nuclear factor erythroid-2-related factor 2 (Nrf2) is a crucial nuclear transcription factor that enters the nucleus when activated and binds to antioxidant response elements to initiates the transcription of a variety of antioxidant genes, and achieves resistance to oxidative stress through this mechanism [34]. Heme oxygenase 1 (HO-1) is an important downstream target molecule of Nrf2 and can catalyze the degradation of heme into biliverdin, CO and iron. These enzymatic products exert strong antioxidant and anti-inflammatory effects. Therefore, activating the Nrf2/HO-1 pathway can reduce oxidative stress damage in the AD brain and alleviate the neurotoxicity of Aβ25-35 [35]. Because palmatine has antioxidant effects and the Nrf2/HO-1 signaling pathway is an important pathway in the antioxidant process, we hypothesized that palmatine achieves its antioxidant effects through the Nrf2/HO-1 signaling pathway.

In this study, we evaluated the neuroprotective effects of palmatine on Aβ25-35-induced AD-like PC12 cells in vitro and established an AD mouse model via the intracranial injection of Aβ25-35 in vivo. Intracranial injections are also well known as a model of AD, and many studies have used this approach for modeling [36,37]. The aim of this study was to explore the influence of palmatine on AD using this model. The possible role of the Nrf2/HO-1 signaling pathway in the effect of palmatine was also analyzed and discussed. Our findings may provide a theoretical basis for palmatine treatment of AD.

## 2. Results

### 2.1. Effects of Palmatine on Cells Viability

**Figure 1:**
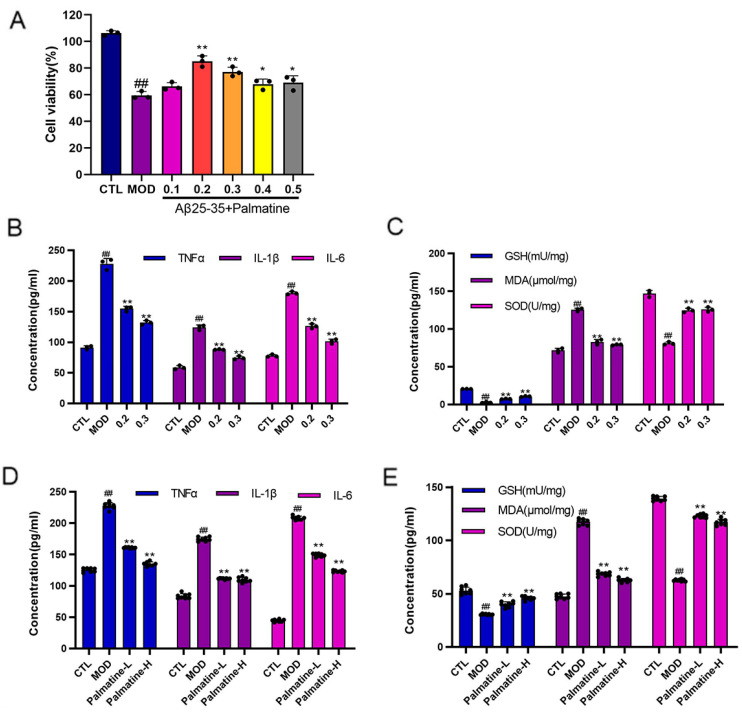
The anti-inflammatory and antioxidant power of palmatine. (**A**) Screening for protective concentrations of palmatine in rat pheochromocytoma cells (PC12) (*n* = 3). (**B**) Anti-inflammatory activity of palmatine in PC12 cells (*n* = 3). (**C**) Antioxidant activity of palmatine in PC12 cells (*n* = 3). (**D**) Anti-inflammatory activity of palmatine in mouse brain tissue (*n* = 8). (**E**) Antioxidant activity of palmatine in mouse brain tissue (*n* = 8). # *p* < 0.05; ## *p* < 0.01 vs. the control group; * *p* < 0.05; ** *p* < 0.01 vs. the model group. CTL: control group; MOD: model group; Palmatine-L: palmatine low-dose group; Palmatine-H: palmatine high-dose group.

Firstly, we determined the concentration and duration of action of Aβ25-35 based on previous studies. PC12 cells were spread evenly over 96-well cell plates and Aβ25-35 (30 μM) was incubated with different concentrations of palmatine (0.1, 0.2, 0.3, 0.4, 0.5 mg/mL) lasting 12 h. As shown in Figure 1A, palmatine has a protective effect on cells. We chose two dose groups of 0.2 mg/mL and 0.3 mg/mL for this study. The model group showed a very significant difference (## *p* < 0.01) compared with the control group, representing a valid model. The palmatine group showed a very significant difference (** *p* < 0.01) compared with the model group at the concentrations of 0.2 and 0.3 mg/mL and 0.4 and 0.5 mg/mL (* *p* < 0.05).

### 2.2. Effects of Palmatine on Pro-Inflammatory Cytokines

We examined the effect of palmatine on the expression of Aβ25-35-induced inflammatory factors in PC12 cells and mice, respectively, using ELISA. As shown in Figure 1B, the TNF-α, IL-1β and IL-6 levels were significantly elevated in PC12 cells induced by Aβ25-35 compared with the control group (*p* < 0.01). Compared with the model group, treatment with palmatine reduced the TNF-α, IL-1β, and IL-6 expression in cells (*p* < 0.01 for all tested cytokines).

Intracranial injection of Aβ25-35 induces neuroinflammation in mice. Neuroinflammation is a trigger for neuronal swollen deformities. As shown in Figure 1D, the levels of inflammatory factors (TNF-α, IL-1β, and IL-6) were elevated in the brains of mice in the model group compared to the control group. The levels of inflammatory factors (TNF-α, IL-1β, and IL-6) were reduced in the group treated with different concentrations of palmatine compared to the model group (*p* < 0.01).

These results indicate that palmatine can effectively reduce the expression of the inflammatory factors TNF-α, IL-1β, and IL-6 in PC12 cells and, in mice, possesses anti-inflammatory properties.

### 2.3. Effects of Palmatine on Oxidative Stress

**Figure 2:**
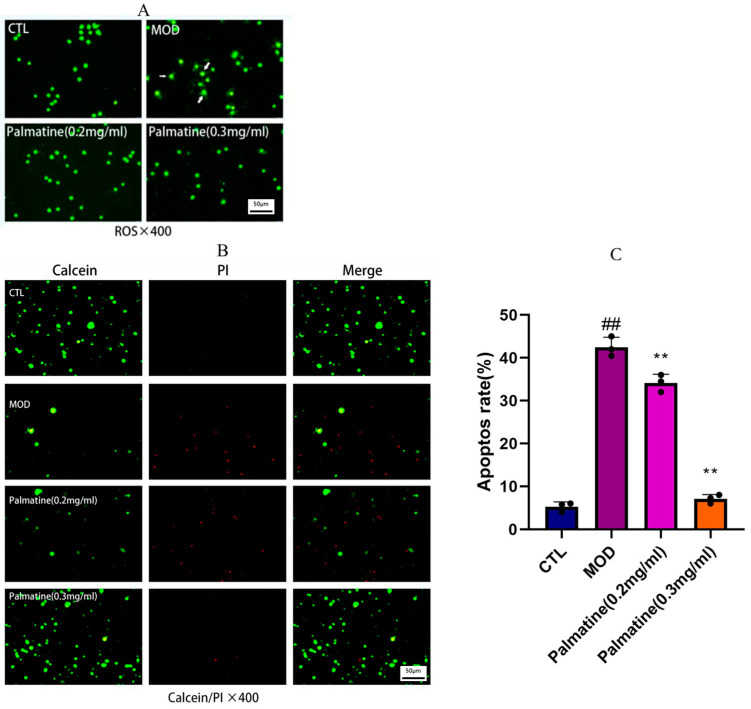
Reactive oxygen and apoptosis staining. (**A**) ROS staining in rat pheochromocytoma cells (*n* = 3). (**B**) Calcein/PI staining in rat pheochromocytoma cells (*n* = 3). (**C**) Percentage of apoptotic cells in the total pc12 cells. # *p* < 0.05; ## *p* < 0.01 vs. the control group; * *p* < 0.05; ** *p* < 0.01 vs. the model group. CTL: control group; MOD: model group.

We also investigated the antioxidant capacity of palmatine. Compared to the control group, exposure to Aβ25-35 led to higher MDA activity and reduced SOD and GSH activity (both *p* < 0.01, as shown in Figure 1C). Treatment with palmatine reduced MDA activity and restored the SOD and GSH activity compared to the control group (*p* < 0.01). Furthermore, the results of immunofluorescence staining indicated that the ROS levels in PC12 cells were decreased by palmatine (Figure 2A).

The results regarding the effect of palmatine on the level of oxidative stress in the brain tissue of mice are as follows, depicted in Figure 1E. (*p* < 0.01). GSH and SOD levels were elevated and MDA levels were reduced in the palmatine-treated group compared to the control group. These changes were reversed in the palmatine-treated group compared to the control group (*p* < 0.01).

In conclusion, palmatine ameliorated Aβ25-35-induced changes in oxidative factor levels and reduced intracellular ROS content via its antioxidant activity.

### 2.4. Effects of Palmatine on Cell Apoptosis

As shown in Figure 2B, most of the PC12 cells in the control group were stained green, with only a very few red apoptotic cells. A large number of cells stained red were observed in the model group, indicating severe apoptosis. This suggests that Aβ25-35 (30 μM) led to apoptosis. Meanwhile, the palmatine 0.2 and 0.3 groups showed a significant reduction in red fluorescence, and the effect of inhibiting apoptosis was better in the palmatine 0.3 group. This indicates that palmatine effectively reduced apoptosis caused by Aβ25-35.

### 2.5. Palmatine Ameliorates Spatial Learning and Memory Deficits in Aβ25-35-Induced AD Mice

**Figure 3:**
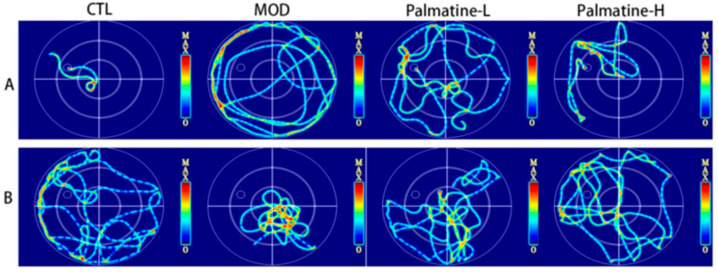
Experimental performance in the Morris water maze test. (**A**) Thermal infrared trajectories of each group in the location navigation experiment (*n* = 3). (**B**) Thermal infrared trajectory of each group in the spatial exploration experiment (*n* = 3). CTL: control group; MOD: model group; Palmatine-L: palmatine low-dose group; Palmatine-H: palmatine high-dose group.

Swimming thermal infrared trajectories and ELT were used to assess spatial learning and memory in the Morris water maze test (Figure 3A). The thermal infrared trajectories of each group of mice in the spatial exploration experiment are displayed in Figure 3B.

**Figure 4:**
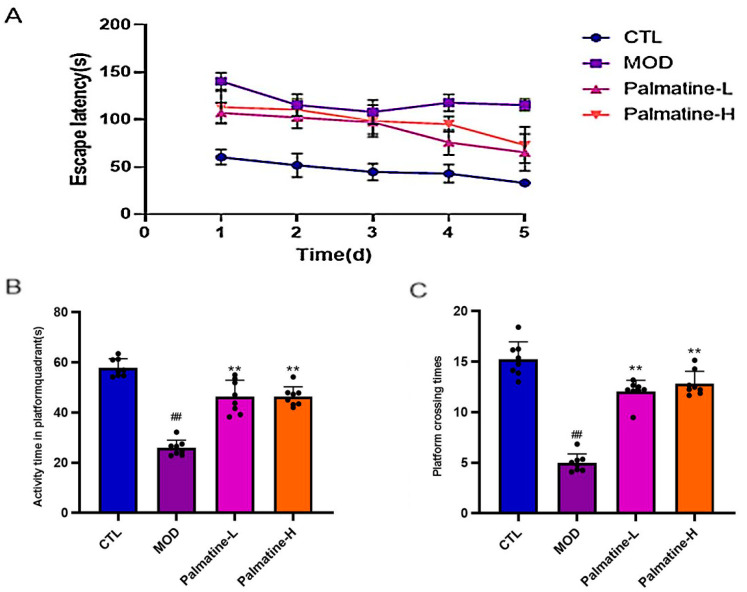
Palmatine improves spatial learning and memory deficits in Alzheimer’s disease mice. (**A**) Escape latency times for each group of mice (*n* = 8). (**B**) Time spent in the target quadrant in the Morris water maze test (*n* = 8). (**C**) The number of times the mice crossed the platform position in the Morris water maze test (*n* = 8). # *p* < 0.05; ## *p* < 0.01 vs. control group; * *p* < 0.05; ** *p* < 0.01 vs. model group. CTL: control group; MOD: model group, Palmatine-L: palmatine low-dose group; Palmatine-H: palmatine high-dose group.

The Morris water maze test results showed that the escape latency of the control, model, Palmatine-L, and Palmatine-H groups gradually decreased during continuous training (Figure 4A). Mice in the model group spent less time in the target quadrant and traversed the virtual platform less frequently compared to the control group (*p* < 0.05) (Figure 4B,C). Both the time spent in the target quadrant and the frequency of crossing the virtual platform were significantly increased in the palmatine group compared to the model group (*p* < 0.05) (Figure 4B,C). The above results suggest that palmatine can improve mouse spatial learning impairment.

### 2.6. Palmatine Reduces Aβ25-35-Induced Neuronal Damage in the Hippocampus of AD Mice

**Figure 5:**
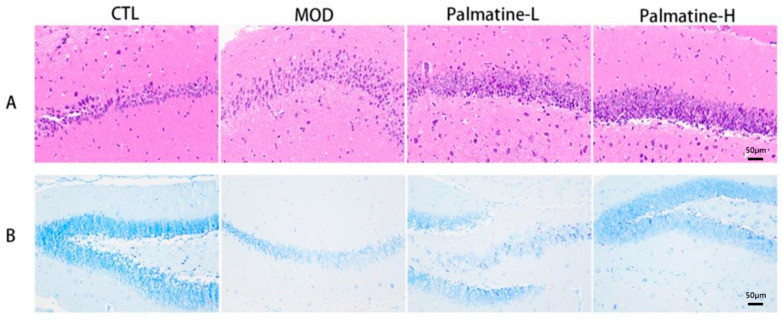
Palmatine reduces hippocampal neuronal damage in Alzheimer’s disease mice. (**A**) Pathological changes in the hippocampal region were detected via HE staining (×200) (*n* = 3). (**B**) Neuronal death in the hippocampal region was detected by means of Nissl staining (×200) (*n* = 3). CTL: control group; MOD: model group; Palmatine-L: palmatine low-dose group; Palmatine-H: palmatine high-dose group.

HE staining demonstrated that the neurons in the hippocampal region of the control group were well arranged, with a normal structure, clear nuclei, obvious nucleoli and abundant cytoplasm, while the model group showed a large number of swollen neurons with a loose structure and solidified nuclei, forming some vacuolar structures. In addition, there were inconspicuous pathological changes in the neurons in the hippocampal region of the palmatine group (Figure 5A).

Next, we investigated the role of palmatine in the survival of hippocampal neurons using Nissl staining (Figure 5B). The number of neurons in the hippocampal region was obvious reduced in the model group in contrast to the control group. Palmatine administration resulted in an augmentation of the amounts of neuronal cells in the hippocampal region. These results indicate that palmatine attenuates the hippocampal neuronal injury caused by Aβ25-35, and it has a protective effect on neuronal cells in the hippocampus.

### 2.7. Effects of Palmatine on the Protein Expression in PC12 Cells and Mouse Hippocampus

**Figure 6:**
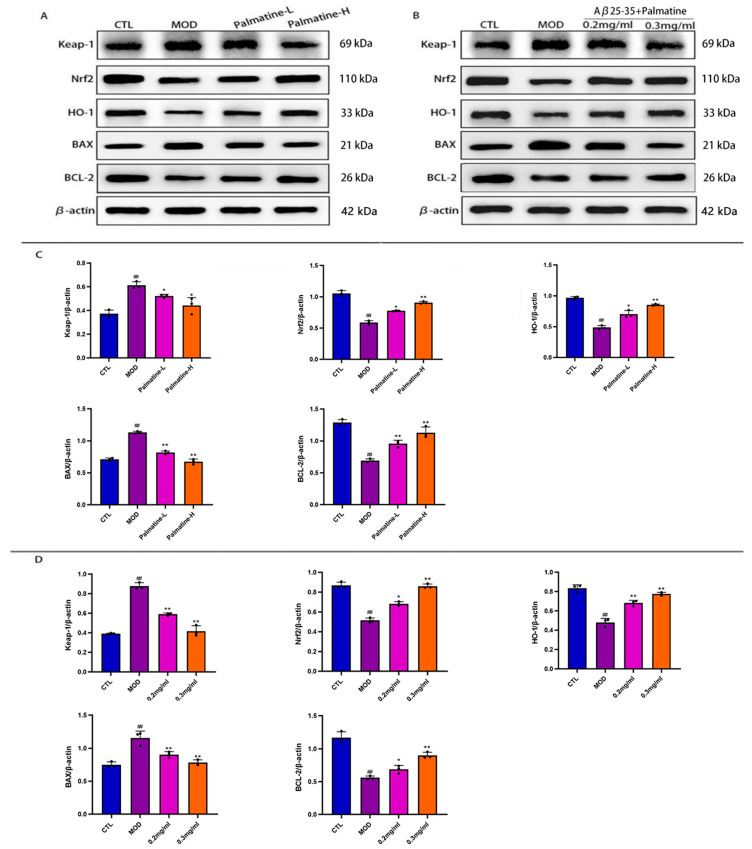
Western blot analysis of Keap-1, Nrf2, HO-1, Bax and Bcl-2 in the hippocampi of Alzheimer’s disease mice and rat pheochromocytoma cells (PC12). (**A**) Protein expressions of Keap-1, Nrf2, HO-1, Bax and Bcl-2 in the hippocampus (*n* = 3). (**B**) Protein expression of Keap-1, Nrf2, HO-1, Bax and Bcl-2 in PC12 cells (*n* = 3). (**C**) Protein expression density of Keap-1, Nrf2, HO-1, Bax and Bcl-2 in the hippocampus (*n* = 3). (**D**) Expression density of Keap-1, Nrf2, HO-1, Bax and Bcl-2 proteins in PC12 cells (*n* = 3). # *p* < 0.05; ## *p* < 0.01 vs. the control group; * *p* < 0.05; ** *p* < 0.01 vs. the model group. CTL: control group; MOD: model group; Palmatine-L: palmatine low-dose group; Palmatine-H: palmatine high-dose group.

Keap1, Nrf2, and HO-1 are all transcription factors related to oxidative stress. The expression of Nrf2 promotes the production of antioxidant factors such as HO-1, which plays an antioxidant role. Keap1 is a negative regulator of Nrf2. It plays an inhibitory role by specifically binding to the sequence of the Neh2 domain of Nrf2 [38]. Apoptosis is closely related to the expression of the proapoptotic protein Bax and the antiapoptotic protein Bcl-2, and the effect of palmatine on apoptosis can be established by assaying them. Western blot analysis demonstrated that the protein expression of Keap-1 and Bax was increased while that of Nrf2, HO-1 and Bcl-2 was decreased in the hippocampus (Figure 6C) and PC12 cells (Figure 6D) of the model group relative to the normal group (*p* < 0.05). In relation to the model group, the protein expression of Keap-1 and Bax was decreased and the protein expression of Nrf2, HO-1 and Bcl-2 was up-regulated in the hippocampus and PC12 cells of palmatine-treated mice (*p* < 0.05). These results suggest that the protective effect of palmatine against AD may be achieved by reducing oxidative stress and decreasing apoptosis, and is related to the Nrf2/HO-1 signaling pathway.

## 3. Discussion

In recent years, the number of patients with Alzheimer’s disease (AD) has increased globally, severely reducing the quality of life of the elderly [39]. Due to the complexity of the causes of Alzheimer’s disease, there is no effective therapeutic drug for AD. Palmatine is a natural alkaloid with a variety of pharmacological activities, but there are fewer studies on its ameliorative effect on AD and its mechanism of action. Therefore, we investigated palmatine’s anti-inflammatory, antioxidation, and antiapoptotic activities and the improvement in learning and memory ability that it induces in mice via both in vivo and in vitro experiments using ELISA, the Morris water maze assay, fluorescent probes, calcein/PI staining, Western blot analysis and other experiments.

In patients with Alzheimer’s disease, antioxidant enzyme activity is diminished, leading to the excessive accumulation of reactive oxygen species, which in turn leads to oxidative damage [40]. As we all know, reactive oxygen species can increase the release of cytochrome-c, activate the expression of caspase proteins and aggravate mitochondrial swelling in the early stages of apoptosis [41]. It was found that Aβ25-35 could raise the levels of ROS and MDA accumulation in PC12 cells while decreasing GSH and SOD activity [42]. In turn, the accumulation of reactive oxygen species in the body leads to an increase in Aβ, and this interaction accelerates the development of AD. The use of antioxidants can be a good way to slow down the progression of AD and is currently receiving a lot of attention [43,44]. Our experimental results show that palmatine significantly restored the antioxidant enzyme activity in PC12 cells.

The deposition of amyloid beta (Aβ) and hyperphosphorylated tau along with glial cell-mediated neuroinflammation are prominent pathogenic hallmarks of AD. Neuroinflammation plays an important role in the pathogenesis of AD. In the early stages of AD pathogenesis, various pro-inflammatory factors (TNF-α, IL-1β, IL-6) activate microglia polarization, thus affecting neurophysiological mechanisms, leading to neuronal synaptic damage and even neuronal degeneration and necrosis [45,46]. There is a lot of research ongoing to improve AD by suppressing inflammation [47,48].In the present study, palmatine inhibited the inflammatory response induced by Aβ25-35, and the reduction in the levels of TNF-α, IL-1β, and IL-6 cytokines validated this idea.

A growing number of studies have shown that apoptosis is related to AD and has been identified as a pathological marker of brain injury [49]. In the present study, the HE staining results show that neuronal atrophy occurs in the model group mice. The calcein/PI staining results also show that Aβ25-35 induced mass apoptosis in PC12 cells, while the use of palmatine improved these conditions and reduced apoptosis. Apoptosis induced by Aβ25-35 may be due to the activation of an intracellular apoptotic cascade, including the activation of Bax in hippocampus neurons. Exposure to Aβ25-35 induced a significant increase in the relative expression of Bax/Bcl-2 [50]. The pro-apoptotic factor Bax and the anti-apoptotic factor Bcl-2 cause mitochondrial membrane permeability in vitro, which is a key pathway of mitochondrial apoptosis [51]. Through Western blot analysis, we found that palmatine can inhibit the expression of Bax, which may be its pathway to ameliorate apoptosis.

Keap1 is a component of the cullin-3 ubiquitin ligase complex that mediates the degradation of Nrf2 as an inhibitor of Nrf2. The transcription factor Nrf2 is tightly regulated in the cytoplasm by Keap1, which plays an important role in the degradation of Nrf2 via the ubiquitin–proteasome pathway, as well as in the protection of the cell against excessive oxidative stress [52]. It is worth mentioning that the NRF2/KEAP1 signaling pathway plays a key role in neurodegenerative diseases [53], inflammatory therapies [54], as well as in a variety of cancerous and non-cancerous diseases, such as pre-eclampsia [55], ovarian cancer [56], breast cancer [57], prostate cancer [58], and traumatic brain injury [59].

Nrf2 is a major protective regulator of antioxidant processes, and the activation of Nrf2 provides neuroprotection against AD [60]. Nrf2 can be activated and bound to antioxidant response elements (ARE) to mediate the expression of peroxiredoxin reductases and second-stage detoxification enzymes such as HO-1 [61]. In addition, HO-1 and its hemoglobin degradation products are highly efficient and sensitive antioxidant enzymes that act as scavengers of ROS and protect cells from oxidative damage and inflammation. In conclusion, the activation of the Nrf2 signaling pathway may be a potential therapeutic target for chronic oxidative stress in neurodegenerative diseases [62]. We found that palmatine can increase the expression of GSH and SOD and reduce the content of intracellular ROS. GSH and SOD expression is regulated by the Nrf2 signaling pathway, and the activation of the Nrf2 signaling pathway can increase the expression of GSH and SOD, thus increasing the antioxidant capacity. Validated by Western blot analysis, we found that palmatine increased the expression of Nrf2 and HO-1 and decreased the expression of Keap-1. These findings suggest that palmatine can inhibit oxidative stress by regulating the Nrf2 signaling pathway.

## 4. Materials and Methods

### 4.1. Materials

Palmatine (Shanghai Yuanye Biotechnology Co., Ltd., Shanghai, China) (3486-67-7); DMEM medium (Gibco, Shanghai, China) (30022.01) and fetal bovine serum (FBS) (Gibco, Shanghai, China) (FB25015); the calcein/PI staining kit (Beyotime, Shanghai, China) (C2015S); 2, 7-dichlorofluorescein diacetate (DCFH-DA) (MCE, Shanghai, China) (HY-D0940); ELISA kits of TNF-α, IL-1β, and IL6 (Beijing Soleibo Technology Co., Ltd., Beijing, China) (SEKM-0034,SEKM-0002,SEKM-0007); SOD, CAT, and GSH detection kits (Beijing Soleibo Technology Co., Ltd., Beijing, China) (BC0175, BC0205, BC1175); the HE staining kit (Beijing Soleibo Technology Co., Ltd., Beijing, China) (G1120); the Nissl Stain Kit (Beijing Soleibo Technology Co., Ltd., Beijing, China) (G1430); Aβ25-35 (MCE, Shanghai, China) (HY-P0128); anti-Nrf2 Recombinant rabbit monoclonal antibody (Huaan Biotechnology Co., Ltd., Hangzhou, China) (HA721432); anti-Keap1 recombinant rabbit monoclonal antibody (Huaan Biotechnology Co., Ltd., Hangzhou, China) (HA721525); anti-heme oxygenase 1 (HO-1) rabbit polyclonal antibody (Huaan Biotechnology Co., Ltd., Hangzhou, China) (ER1802-73); anti-Bax recombinant rabbit monoclonal antibody (Huaan Biotechnology Co., Ltd., Hangzhou, China) (ET1603-34); anti-Bcl-2 recombinant rabbit monoclonal antibody (Huaan Biotechnology Co., Ltd., Hangzhou, China) (ET1702-53); β-actin recombinant rabbit monoclonal antibody (Wanlei Biotechnology Co., Ltd., Shenyang, China) (WL01372); HRP-goat anti-rabbit IgG (H + L) (Wanlei Biotechnology Co., Ltd., Shenyang, China) (WLA023).

### 4.2. Cell Culture and Treatment

PC12 cells were obtained from the Chinese Academy of Sciences. PC12 cells were cultured in RPMI-1640 medium which contained 10% heat-inactivated FBS and 1% penicillin/streptomycin. The ambient temperature was 37 °C, and the CO_2_ content was 5%.

### 4.3. Cell Viability Assay

First of all, PC12 cells were inoculated onto a 96-well culture plate, and the density of cells was adjusted to 1×10^4^ cells/well. The cells were allowed to grow for 12 h at 37 °C. The experiment was divided into the control group, the model group (30 μM Aβ25-35), and the Aβ25-35 (30 μM) and different concentrations of palmatine (0.1, 0.2, 0.3, 0.4, 0.5 mg/mL) group. Then, each group was cultured with PC12 cells for 12 h. Finally, cell viability was tested by CCK-8. We added 10 μL of CCK-8 per well and performed measurements at a wavelength of 450 nm using a microplate reader after 2 h at 37 °C [63]. The two groups with the best cellular activity were selected from the Aβ25-35 (30 μM) and different concentrations of palmatine (0.1, 0.2, 0.3, 0.4, 0.5 mg/mL) group (Aβ25-35 + palmatine), along with the control group and the model group, for subsequent experiments.

The calculation of cell viability (%) was as follows:

Cell viability (100%) = (Adrug − Ablank)/(Acontrol − Ablank) × 100%

### 4.4. AD Model Construction and Treatment

**Figure 7:**
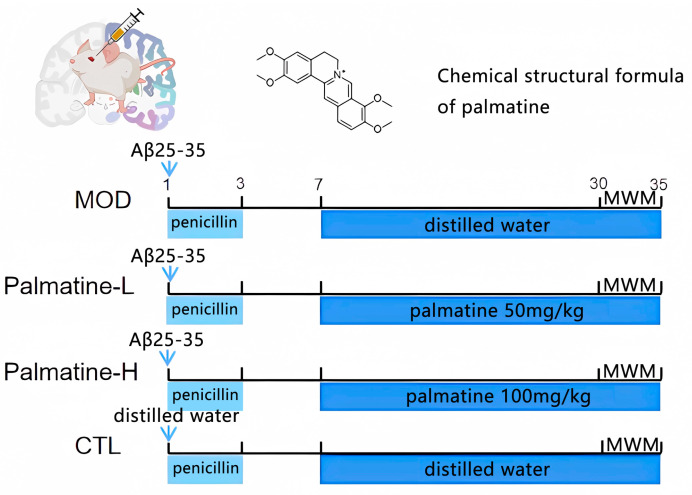
Administration flow chart. Modeling was performed via the craniosurgical method, and 1.5 μL of Aβ25-35 (2 μg/μL) was injected. From the 7th to the 35th postoperative day, 50 mg/kg palmatine was administered via gavage to the palmatine low-dose group and 100 mg/kg palmatine was administered to the palmatine high-dose group. CTL: control group; MOD: model group; Palmatine-L: palmatine low-dose group; Palmatine-H: palmatine high-dose group.

The mice were divided into 4 groups: the normal control group, the model group (Aβ25-35), the palmatine low-dose group (Palmatine-L: Aβ25-35 + palmatine 50 mg/kg), and the palmatine high-dose group (Palmatine-H: Aβ25-35 + palmatine 100 mg/kg). There were 10 mice in each group. All groups except for the control mice were modelled via the injection of 1.5 μL of Aβ25-35 (2 μg/μL). Mice in the control group were administered 1.5 μL of normal saline instead of Aβ25-35. On the seventh postoperative day, 50 mg/kg palmatine was administered via gavage to the palmatine low-dose group, and 100 mg/kg palmatine was administered to the palmatine high dose group, and an equal dose of distilled water was administered via gavage to the model and blank control groups. Each mouse was administered 0.2 mL via gavage, and its body weight was about 20 g. Therefore, we configured the concentration of palmatine in the low-dose group to be 5 mg/mL and that of palmatine in the high-dose group to be 10 mg/mL. Mice were modelled as follows: firstly, they were anesthetized via the intraperitoneal injection of 0.4% pentobarbital sodium (0.2 mL), and then the mouse was fixed on a stereotactic brain positioner and the skin on its head and neck was incised. The CA1 area of the bilateral hippocampus was used as the delivery site. Bregmatic fontanel was the base, and the lateral ventricle was located 2 mm behind and 1.5 mm away from the sagittal suture. A 2.5 mm deep hole was drilled into the skull surface using a microcranial drill, and 1.5 µL of Aβ25-35 (2 µg/µL) was injected at a constant rate of 0.2 µL per minute using a microinjection needle [64]. We left the needle in for 1 min and slowly withdrew it. The intraperitoneal injection of penicillin was needed three days after surgery to prevent infection (Figure 7).

We used SPF-grade 7–8-week-old male ICR mice, weighing 20–22 g, purchased from Changchun Yisi Experimental Animal Technology Co., Ltd. (Changchun, China). The animals were kept at room temperature under standard laboratory conditions. The temperature was 23 ± 2 °C, and the humidity was 60 ± 10%. During the experiment, the mice were allowed to eat and drink freely. All animal procedures were performed in accordance with the National Institutes of Health and institutional guidelines for the humane care of animals, and approved by the Animal Care Committee of Jilin Agricultural University (2016–01-Permit number: ECLA-JLAU 2016–016) (No.2020 11 18 001).

### 4.5. Morris Water Maze Test

The Morris water maze test consisted of a circular pool with a diameter of 1.2 m. The pool was filled with water (23–25 °C). A dark and quiet environment was maintained in the water maze. The pool was divided equally into four quadrants, while the escape platform was randomly placed in the center of one quadrant. The escape times and trajectories of mice were recorded using software and video tracking systems.

Mice swam freely in the water maze from any starting position to find the platform. If it failed to find the platform, the mouse was guided to stand on the platform for 10 s and was trained 4 times a day for 5 days [65].

The Morris water maze test was performed on the last five days of the experiment. First, the avoidance latency of the mice was recorded in the positional navigation test, and the time it took for the mice to find the platform within 60 s was recorded, followed by the spatial exploration test, in which the mice’s movements were observed over a period of 60 s [66].

### 4.6. Evaluation of Oxidative Stress

The overproduction of reactive oxygen species is one of the hallmarks of excessive oxidative stress in cells. ROS production in PC12 cells was measured using a fluorescent probe (DCFH-DA). Briefly, PBS was used to wash each cell twice. Then, 1 mL of DCFH-DA solution (final concentration 10 μM) was added to each well and incubated in a CO_2_ incubator at 37 °C for 30 min. The cells were then washed with PBS three times to remove the DCFH-DA which did not enter the cells. Cellular ROS content assessment was performed using a fluorescent microscope (Olympus, Tokyo, Japan) [67].

### 4.7. Calcein/PI Staining

In order to test the effect of palmatine on the apoptotic cell death situation, cells were stained with calcein/PI staining and fluorescence microscopy was performed on the stained cells (Olympus, Tokyo, Japan) [68]. Calcein AM was used to stain live cells with green fluorescence and propidium iodide (PI) was used to stain dead cells with red fluorescence.

### 4.8. Enzyme-Linked Immunosorbent Assay (ELISA)

To further investigate the effects of palmatine on the levels of inflammatory and oxidative factors in mice in vivo and in PC12 cells, we used an ELISA assay to detect their levels. To detect the levels of the inflammatory cytokines TNF-α, IL-1β and IL-6 in the PC12 cell supernatant and mouse serum, the assays were performed on the basis of the ELISA kit manufacturer’s protocol [69]. Moreover, chemical analysis kits were used to determine the contents of GSH, MDA and SOD in PC12 cells and mouse brain tissue [70,71]. The steps were as follows: we dissected the mice and collected the brain tissue. The brain tissue was homogenized and centrifuged at 4000 r/min for 10 min, and then the levels of GSH, SOD and MDA were measured using the appropriate kits.

### 4.9. Staining of the Hippocampus in Mice

In order to observe the cell morphology and apoptosis in the mouse hippocampus, we sectioned and stained the mouse hippocampus as follows. We took the brain tissue of mice and fixed it with 4% formaldehyde. After dehydration and transparency, paraffin-embedded tissue was sectioned into 5 μm thick sections. The tissue sections were dewaxed with xylenes, and then rehydrated with gradient alcohol. Tissue sections were stained with HE and Nissl dye [69].

### 4.10. Western Blot Analysis

The effect of palmatine on protein expression in mouse brain was explored using Western blot analysis. We took 0.1 g–0.2 g of mouse brain tissue, ground it on ice with a grinding rod and added 0.5 mL of lysate, ground it to a homogenous state, and centrifuged it at 12,000 rpm for 15 min at 4 °C. After centrifugation, we took the supernatant, determined the concentration of proteins, adjusted the concentration of each tube and added the loading buffer, and then boiled for ten minutes to obtain the protein samples. A 10% sodium dodecyl sulfate-polyacrylamide gel electrophoresis (SDS-PAGE) was applied to the protein sample and then transferred to nitrocellulose membranes. Membranes were blocked with 5% skimmed milk in TBST and then incubated overnight at 4 °C with the primary antibodies Nrf2 (1:1000), Keap1 (1:1000), HO-1 (1:1000), BAX (1:1000), BCL-2 (1:1000), and β-actin (1:1000) [72]. The membranes were washed 3 times with TBST at the end of the primary antibody treatment, then we added the secondary antibodies and incubate the membranes at room temperature for 2 h.

### 4.11. Statistical Analysis

All figures were created using Graphpad Prism 8.0.2. Statistical analysis was conducted using SPSS19.0 software. All data are expressed as the average mean ± SEM. Data were analyzed via one-way or two-way ANOVA with Tukey’s test for comparison between multiple groups. A value of *p* < 0.05 was considered significant.

## 5. Conclusions

To sum up, we demonstrate that palmatine is neuroprotective against Aβ25-35-induced neurotoxicity by regulating the Nrf2/HO-1 pathway in PC12 cells and mice. Our results suggest that palmatine may be a drug candidate with anti-neuroinflammatory properties for the treatment of neurodegenerative diseases.

## Data Availability

The research data used to support the findings of this study are included in the article.

## References

[1] Wang L., Zhou B.Q., Li Y.H., Jiang Q.Q., Cong W.H., Chen K.J., Wen X.M., Wu Z.Z. (2023). Lactoferrin modification of berberine nanoliposomes enhances the neuroprotective effects in a mouse model of Alzheimer’s disease. Neural Regen. Res..

[2] Han J., Zhang H., Zhang Y., Zhang Z., Yu M., Wang S., Han F. (2022). Lingguizhugan decoction protects PC12 cells against Abeta25-35-induced oxidative stress and neuroinflammation by modulating NF-kappaB/MAPK signaling pathways. J. Ethnopharmacol..

[3] Barage S.H., Sonawane K.D. (2015). Amyloid cascade hypothesis: Pathogenesis and therapeutic strategies in Alzheimer’s disease. Neuropeptides.

[4] Ding M.R., Qu Y.J., Hu B., An H.M. (2022). Signal pathways in the treatment of Alzheimer’s disease with traditional Chinese medicine. Biomed. Pharmacother..

[5] Cai T., Che H., Yao T., Chen Y., Huang C., Zhang W., Du K., Zhang J., Cao Y., Chen J. (2011). Manganese induces tau hyperphosphorylation through the activation of ERK MAPK pathway in PC12 cells. Toxicol. Sci..

[6] Wang X., Tang T., Zhai M., Ge R., Wang L., Huang J., Zhou P. (2020). Ling-Gui-Zhu-Gan Decoction Protects H9c2 Cells against H_2_O_2_-Induced Oxidative Injury via Regulation of the Nrf2/Keap1/HO-1 Signaling Pathway. Evid. Based Complement. Altern. Med..

[7] Zeng Q., Li L., Siu W., Jin Y., Cao M., Li W., Chen J., Cong W., Ma M., Chen K. (2019). A combined molecular biology and network pharmacology approach to investigate the multi-target mechanisms of Chaihu Shugan San on Alzheimer’s disease. Biomed. Pharmacother..

[8] Ji Q., Zhu F., Liu X., Li Q., Su S.B. (2015). Recent Advance in Applications of Proteomics Technologies on Traditional Chinese Medicine Research. Evid. Based Complement. Altern. Med..

[9] Zeng J., Pei H., Wu H., Chen W., Du R., He Z. (2023). Palmatine attenuates LPS-induced neuroinflammation through the PI3K/Akt/NF-κB pathway. J. Biochem. Mol. Toxicol..

[10] Committee S.P. (2020). Pharmacopoeia of the Peoples Republic of China.

[11] He Z., Yu H., Wu H., Su L., Shi K., Zhao Y., Zong Y., Chen W., Du R. (2022). Antidepressant effects of total alkaloids of Fibraurea recisa on improving corticosterone-induced apoptosis of HT-22 cells and chronic unpredictable mild stress-induced depressive-like behaviour in mice. Pharm. Biol..

[12] Tarabasz D., Kukula-Koch W. (2020). Palmatine: A review of pharmacological properties and pharmacokinetics. Phytother. Res..

[13] Zhou X., Lin X., Xiong Y., Jiang L., Li W., Li J., Wu L. (2016). Chondroprotective effects of palmatine on osteoarthritis in vivo and in vitro: A possible mechanism of inhibiting the Wnt/β-catenin and Hedgehog signaling pathways. Int. Immunopharmacol..

[14] Zheng J.Y., Li G.F., He Z.M., Zhu H.Y., Zhao Y., Gao Y.G., Yang H., Zhang L.X. (2017). Pharmacokinetics and oral bioavailability of palmatine and jatrorrhizine in Huangteng in rats. Zhongguo Zhong Yao Za Zhi.

[15] Wang S., Ma Y., Huang Y., Hu Y., Huang Y., Wu Y. (2022). Potential bioactive compounds and mechanisms of Fibraurea recisa Pierre for the treatment of Alzheimer’s disease analyzed by network pharmacology and molecular docking prediction. Front. Aging Neurosci..

[16] Cheng D., Liu P., Wang Z. (2022). Palmatine attenuates the doxorubicin-induced inflammatory response, oxidative damage and cardiomyocyte apoptosis. Int. Immunopharmacol..

[17] Lee W.C., Kim J.K., Kang J.W., Oh W.Y., Jung J.Y., Kim Y.S., Jung H.A., Choi J.S., Lee S.M. (2010). Palmatine attenuates D-galactosamine/lipopolysaccharide-induced fulminant hepatic failure in mice. Food Chem. Toxicol..

[18] Wang L., Li M., Zhu C., Qin A., Wang J., Wei X. (2022). The protective effect of Palmatine on depressive like behavior by modulating microglia polarization in LPS-induced mice. Neurochem. Res..

[19] Pereira J.F., de Sousa Neves J.C., Fonteles A.A., Bezerra J.R., Pires R.C., da Silva A.T.A., Lima F.A.V., Neves K.R.T., Oriá R.B., de Barros Viana G.S. (2023). Palmatine, a natural alkaloid, attenuates memory deficits and neuroinflammation in mice submitted to permanent focal cerebral ischemia. J. Neuroimmunol..

[20] Tang C., Hong J., Hu C., Huang C., Gao J., Huang J., Wang D., Geng Q., Dong Y. (2021). Palmatine Protects against Cerebral Ischemia/Reperfusion Injury by Activation of the AMPK/Nrf2 Pathway. Oxidative Med. Cell. Longev..

[21] Chaves S.K.M., Afzal M.I., Islam M.T., Hameed A., Da Mata A., Da Silva Araújo L., Ali S.W., Rolim H.M.L., De Medeiros M., Costa E.V. (2020). Palmatine antioxidant and anti-acetylcholinesterase activities: A pre-clinical assessment. Cell. Mol. Biol. (Noisy-le-grand).

[22] Xie D., Deng T., Zhai Z., Sun T., Xu Y. (2022). The cellular model for Alzheimer’s disease research: PC12 cells. Front. Mol. Neurosci..

[23] Chalatsa I., Arvanitis D.A., Mikropoulou E.V., Giagini A., Papadopoulou-Daifoti Z., Aligiannis N., Halabalaki M., Tsarbopoulos A., Skaltsounis L.A., Sanoudou D. (2018). Beneficial Effects of Sideritis scardica and Cichorium spinosum against Amyloidogenic Pathway and Tau Misprocessing in Alzheimer’s Disease Neuronal Cell Culture Models. J. Alzheimers Dis..

[24] Zhang Y.Y., Bao H.L., Dong L.X., Liu Y., Zhang G.W., An F.M. (2021). Silenced lncRNA H19 and up-regulated microRNA-129 accelerates viability and restrains apoptosis of PC12 cells induced by Aβ(25-35) in a cellular model of Alzheimer’s disease. Cell Cycle.

[25] Gao L., Zhou F., Wang K.X., Zhou Y.Z., Du G.H., Qin X.M. (2020). Baicalein protects PC12 cells from Aβ(25)(-)(35)-induced cytotoxicity via inhibition of apoptosis and metabolic disorders. Life Sci..

[26] Cheng W., Chen W., Wang P., Chu J. (2018). Asiatic acid protects differentiated PC12 cells from Aβ(25–35)-induced apoptosis and tau hyperphosphorylation via regulating PI3K/Akt/GSK-3β signaling. Life Sci..

[27] Cui J., Wang J., Zheng M., Gou D., Liu C., Zhou Y. (2017). Ginsenoside Rg2 protects PC12 cells against β-amyloid(25–35)-induced apoptosis via the phosphoinositide 3-kinase/Akt pathway. Chem. Biol. Interact..

[28] Askarova S., Tsoy A., Shalakhmetova T., Lee J.C. (2012). Effects of Amyloid Beta Peptide on Neurovascular Cells. Cent. Asian J. Glob. Health.

[29] Naldi M., Fiori J., Pistolozzi M., Drake A.F., Bertucci C., Wu R., Mlynarczyk K., Filipek S., De Simone A., Andrisano V. (2012). Amyloid β-peptide 25–35 self-assembly and its inhibition: A model undecapeptide system to gain atomistic and secondary structure details of the Alzheimer’s disease process and treatment. ACS Chem. Neurosci..

[30] Arispe N., Rojas E., Pollard H.B. (1993). Alzheimer disease amyloid beta protein forms calcium channels in bilayer membranes: Blockade by tromethamine and aluminum. Proc. Natl. Acad. Sci. USA.

[31] Changhong K., Peng Y., Yuan Z., Cai J. (2021). Ginsenoside Rb1 protected PC12 cells from Aβ(25–35)-induced cytotoxicity via PPARγ activation and cholesterol reduction. Eur. J. Pharmacol..

[32] Limón I.D., Díaz A., Mendieta L., Chamorro G., Espinosa B., Zenteno E., Guevara J. (2009). Amyloid-beta(25–35) impairs memory and increases NO in the temporal cortex of rats. Neurosci. Res..

[33] Tadokoro K., Ohta Y., Inufusa H., Loon A.F.N., Abe K. (2020). Prevention of Cognitive Decline in Alzheimer’s Disease by Novel Antioxidative Supplements. Int. J. Mol. Sci..

[34] Brandes M.S., Gray N.E. (2020). NRF2 as a Therapeutic Target in Neurodegenerative Diseases. ASN Neuro.

[35] Xu J., Zhou L., Weng Q., Xiao L., Li Q. (2019). Curcumin analogues attenuate Aβ(25-35)-induced oxidative stress in PC12 cells via Keap1/Nrf2/HO-1 signaling pathways. Chem. Biol. Interact..

[36] Stepanichev M., Lazareva N.A., Onufriev M.V., Mitrokhina O.S., Moiseeva Yu V., Gulyaeva N.V. (1998). Effects of doses of fragment (25–35) of beta-amyloid peptide on behavior in rats. Neurosci. Behav. Physiol..

[37] Pike C.J., Burdick D., Walencewicz A.J., Glabe C.G., Cotman C.W. (1993). Neurodegeneration induced by beta-amyloid peptides in vitro: The role of peptide assembly state. J. Neurosci..

[38] Yan J., Pang Y., Zhuang J., Lin H., Zhang Q., Han L., Ke P., Zhuang J., Huang X. (2019). Selenepezil, a Selenium-Containing Compound, Exerts Neuroprotective Effect via Modulation of the Keap1-Nrf2-ARE Pathway and Attenuates Aβ-Induced Cognitive Impairment in Vivo. ACS Chem. Neurosci..

[39] Landeiro F., Walsh K., Ghinai I., Mughal S., Nye E., Wace H., Roberts N., Lecomte P., Wittenberg R., Wolstenholme J. (2018). Measuring quality of life of people with predementia and dementia and their caregivers: A systematic review protocol. BMJ Open.

[40] Khan T.A., Hassan I., Ahmad A., Perveen A., Aman S., Quddusi S., Alhazza I.M., Ashraf G.M., Aliev G. (2016). Recent Updates on the Dynamic Association Between Oxidative Stress and Neurodegenerative Disorders. CNS Neurol. Disord. Drug Targets.

[41] Dong Q., Li Z., Zhang Q., Hu Y., Liang H., Xiong L. (2022). Astragalus mongholicus Bunge (Fabaceae): Bioactive Compounds and Potential Therapeutic Mechanisms Against Alzheimer’s Disease. Front. Pharmacol..

[42] Cui W., Sun C., Ma Y., Wang S., Wang X., Zhang Y. (2020). Inhibition of TLR4 Induces M2 Microglial Polarization and Provides Neuroprotection via the NLRP3 Inflammasome in Alzheimer’s Disease. Front. Neurosci..

[43] Goshtasbi H., Pakchin P.S., Movafeghi A., Barar J., Castejon A.M., Omidian H., Omidi Y. (2022). Impacts of oxidants and antioxidants on the emergence and progression of Alzheimer’s disease. Neurochem. Int..

[44] Wojsiat J., Zoltowska K.M., Laskowska-Kaszub K., Wojda U. (2018). Oxidant/Antioxidant Imbalance in Alzheimer’s Disease: Therapeutic and Diagnostic Prospects. Oxidative Med. Cell. Longev..

[45] Hu Y.R., Xing S.L., Chen C., Shen D.Z., Chen J.L. (2021). Codonopsis pilosula Polysaccharides Alleviate Aβ1-40-Induced PC12 Cells Energy Dysmetabolism via CD38/NAD+ Signaling Pathway. Curr. Alzheimer Res..

[46] Shi K., Chen L., Chen L., Tan A., Xie G., Long Q., Ning F., Lan Z., Wang P. (2022). Epimedii Folium and Curculiginis Rhizoma ameliorate lipopolysaccharides-induced cognitive impairment by regulating the TREM2 signaling pathway. J. Ethnopharmacol..

[47] Chen C., Liao J., Xia Y., Liu X., Jones R., Haran J., McCormick B., Sampson T.R., Alam A., Ye K. (2022). Gut microbiota regulate Alzheimer’s disease pathologies and cognitive disorders via PUFA-associated neuroinflammation. Gut.

[48] Wang C., Chen S., Guo H., Jiang H., Liu H., Fu H., Wang D. (2022). Forsythoside A Mitigates Alzheimer’s-like Pathology by Inhibiting Ferroptosis-mediated Neuroinflammation via Nrf2/GPX4 Axis Activation. Int. J. Biol. Sci..

[49] Shi M., Sun F., Wang Y., Kang J., Zhang S., Li H. (2020). CGA restrains the apoptosis of Aβ(25–35)-induced hippocampal neurons. Int. J. Neurosci..

[50] Xie L.Y., Yang Z., Wang Y., Hu J.N., Lu Y.W., Zhang H., Jiang S., Li W. (2022). 1-O-Actylbritannilactone Ameliorates Alcohol-Induced Hepatotoxicity through Regulation of ROS/Akt/NF-kappaB-Mediated Apoptosis and Inflammation. ACS Omega.

[51] Wang C., Cai X., Hu W., Li Z., Kong F., Chen X., Wang D. (2019). Investigation of the neuroprotective effects of crocin via antioxidant activities in HT22 cells and in mice with Alzheimer’s disease. Int. J. Mol. Med..

[52] Bian Y., Chen Y., Wang X., Cui G., Ung C.O.L., Lu J.H., Cong W., Tang B., Lee S.M. (2021). Oxyphylla A ameliorates cognitive deficits and alleviates neuropathology via the Akt-GSK3beta and Nrf2-Keap1-HO-1 pathways in vitro and in vivo murine models of Alzheimer’s disease. J. Adv. Res..

[53] Phukan B.C., Roy R., Paul R., Mazumder M.K., Nath J., Bhattacharya P., Borah A. (2023). Traversing through the cell signaling pathways of neuroprotection by betanin: Therapeutic relevance to Alzheimer’s Disease and Parkinson’s Disease. Metab. Brain Dis..

[54] Yang S., Li F., Lu S., Ren L., Bian S., Liu M., Zhao D., Wang S., Wang J. (2022). Ginseng root extract attenuates inflammation by inhibiting the MAPK/NF-κB signaling pathway and activating autophagy and p62-Nrf2-Keap1 signaling in vitro and in vivo. J. Ethnopharmacol..

[55] Tossetta G., Fantone S., Piani F., Crescimanno C., Ciavattini A., Giannubilo S.R., Marzioni D. (2023). Modulation of NRF2/KEAP1 Signaling in Preeclampsia. Cells.

[56] Tossetta G., Fantone S., Montanari E., Marzioni D., Goteri G. (2022). Role of NRF2 in Ovarian Cancer. Antioxidants.

[57] Ghareghomi S., Habibi-Rezaei M., Arese M., Saso L., Moosavi-Movahedi A.A. (2022). Nrf2 Modulation in Breast Cancer. Biomedicines.

[58] Tossetta G., Fantone S., Marzioni D., Mazzucchelli R. (2023). Cellular Modulators of the NRF2/KEAP1 Signaling Pathway in Prostate Cancer. Front. Biosci..

[59] Abdul-Muneer P.M. (2023). Nrf2 as a Potential Therapeutic Target for Traumatic Brain Injury. J. Integr. Neurosci..

[60] Saad El-Din S., Rashed L., Medhat E., Emad Aboulhoda B., Desoky Badawy A., Mohammed ShamsEldeen A., Abdelgwad M. (2020). Active form of vitamin D analogue mitigates neurodegenerative changes in Alzheimer’s disease in rats by targeting Keap1/Nrf2 and MAPK-38p/ERK signaling pathways. Steroids.

[61] Wang Q., Yang Z., Zhuang J., Zhang J., Shen F., Yu P., Zhong H., Feng F. (2022). Antiaging function of Chinese pond turtle (*Chinemys reevesii*) peptide through activation of the Nrf2/Keap1 signaling pathway and its structure-activity relationship. Front. Nutr..

[62] Sha J.Y., Li J.H., Zhou Y.D., Yang J.Y., Liu W., Jiang S., Wang Y.P., Zhang R., Di P., Li W. (2021). The p53/p21/p16 and PI3K/Akt signaling pathways are involved in the ameliorative effects of maltol on D-galactose-induced liver and kidney aging and injury. Phytother. Res..

[63] Ding J., Jia W., Cui Y., Jin J., Zhang Y., Xu L., Liu Y. (2020). Anti-angiogenic effect of a chemically sulfated polysaccharide from Phellinus ribis by inhibiting VEGF/VEGFR pathway. Int. J. Biol. Macromol..

[64] Zhang M., Chen W., Zong Y., Shi K., Li J., Zeng F., He Z., Du R. (2020). Cognitive-enhancing effects of fibrauretine on Abeta(1-42)-induced Alzheimer’s disease by compatibilization with ginsenosides. Neuropeptides.

[65] Xie C., Tang H., Liu G., Li C. (2022). Molecular mechanism of Epimedium in the treatment of vascular dementia based on network pharmacology and molecular docking. Front. Aging Neurosci..

[66] Shen H., He Z., Pei H., Zhai L., Guan Q., Wang G. (2022). Aurantiamide promotes M2 polarization of microglial cells to improve the cognitive ability of mice with Alzheimer’s disease. Phytother. Res..

[67] Zhao Y., Wang Y., Zhang M., Gao Y., Yan Z. (2021). Protective Effects of Ginsenosides (20R)-Rg3 on H_2_O_2_-Induced Myocardial Cell Injury by Activating Keap-1/Nrf2/HO-1 Signaling Pathway. Chem. Biodivers..

[68] Lu M.D., Li H., Nie J.H., Li S., Ye H.S., Li T.T., Wu M.L., Liu J. (2022). Dual Inhibition of BRAF-MAPK and STAT3 Signaling Pathways in Resveratrol-Suppressed Anaplastic Thyroid Cancer Cells with BRAF Mutations. Int. J. Mol. Sci..

[69] Du Q., Zhu X., Si J. (2020). Angelica polysaccharide ameliorates memory impairment in Alzheimer’s disease rat through activating BDNF/TrkB/CREB pathway. Exp. Biol. Med. (Maywood).

[70] AlKahtane A.A., Ghanem E., Bungau S.G., Alarifi S., Ali D., AlBasher G., Alkahtani S., Aleya L., Abdel-Daim M.M. (2020). Carnosic acid alleviates chlorpyrifos-induced oxidative stress and inflammation in mice cerebral and ocular tissues. Environ. Sci. Pollut. Res. Int..

[71] He G., Zhao Q., Zhao Y., Zong Y., Gu S., Li M., Li R., Sun J. (2022). Deer antler based active ingredients have protective effects on LPS/d-GalN-induced acute liver injury in mice through MAPK and NF-kappaB signalling pathways. Pharm. Biol..

[72] Li N., Wen L., Wang F., Li T., Zheng H., Wang T., Qiao M., Huang X., Song L., Bukyei E. (2022). Alleviating effects of pea peptide on oxidative stress injury induced by lead in PC12 cells via Keap1/Nrf2/TXNIP signaling pathway. Front. Nutr..

